# Clinical characteristics and risk factors of patients with eosinophilic fasciitis associated with pleural effusion

**DOI:** 10.1038/s41598-023-32678-2

**Published:** 2023-04-03

**Authors:** Zhifeng Chen, Binaya Wasti, Yulin Shang, Aijun Jia, Xudong Xiang, Ruoyun Ouyang

**Affiliations:** 1 Department of Respiratory Medicine, The Second Xiangya Hospital, Central South University, 139 Middle Renmin Road, Changsha, 410011 Hunan China; 2 Ophthalmology and Otorhinolaryngology, Zigui County Traditional Chinese Medicine Hospital, 30 Pinghu Avenue, Zigui, 443600 Hubei China; 3 Department of the Third Emergency of Yuelushan Hospital District, Hunan Provincial People’s Hospital, No.90 Pingchuan Road, Changsha, 410006 Hunan China; 4 Department of Emergency, The Second Xiangya Hospital, Central South University, 139 Middle Renmin Road, Changsha, 410011 Hunan China

**Keywords:** Respiration, Skin diseases

## Abstract

To investigate the risk factors of eosinophilic fasciitis (EF) associated with pleural effusion (PE). A retrospective analysis was performed on 22 patients with EF diagnosed by skin biopsy in our hospital, and they were divided into EF-PE and EF according to chest computed tomography examination. The clinical characteristics, clinical manifestations, comorbidities and laboratory test indicators of the two groups were collected and compared, and the risk factors for occurring PE in patients with EF were determined by multivariate logistic regression analysis. Among 22 patients with EF, 8 had PE. The age, course of disease, incidence of fever, weight loss, cough and shortness of breath, pulmonary infection, hypothyroidism, hydronephrosis and kidney stone, swelling rate of small vascular endothelial cells, consolidation shadows, C-reactive protein and thyroid stimulating hormone in EF-PE group were higher than those in EF group, while free triiodothyronine and thyroxine were lower than those in EF group. Age, fever, shortness of breath, C-reactive protein, ESR, thyroid stimulating hormone, pulmonary infection, hypothyroidism, hydronephrosis, kidney stones, swollen small vascular endothelial cells and chest CT consolidation shadows were identified as risk factors for happening PE in patients with EF, while free triiodothyronine and free thyroxine were identified as protective factors against PE in patients with EF. The incidence of EF-PE was 36.36% in this study. Advanced age, high C-reactive protein, ESR, thyroid stimulating hormone, incidence of fever, shortness of breath, pulmonary infection, hydronephrosis, kidney stones, swollen small vascular endothelial cells, chest CT consolidation shadows, and low free triiodothyronine and thyroxine suggest that patients with EF are significantly at increased risk of PE.

## Introduction

Eosinophilic Fasciitis (EF), also known as Shulman syndrome, first reported by Shulman in 1974, which is characterized by progressive sclerosclerosis of symmetrical limbs, fascial hyperplasia, peripheral eosinophilia, hypergammaglobulinemia, joint pain, and increased erythrocyte sedimentation rate (ESR) with lymphocytes and plasmocytes infiltration. Approximately 53–67% of patients are sensitive to oral corticosteroid therapy^1–3^. EF can cause haematological diseases such as aplastic anemia, T-cell lymphoma, Hodgkin’s disease, multiple myeloma, myelodysplastic syndrome, and autoimmune diseases such as Hashimoto’s thyroiditis, systemic lupus erythematosus, and glomerulonephritis. It can also cause pneumonia and pleural diseases^4,5^. Braga et al.^6^ reported that an EF patient presented symptoms of dyspnea, lumbago and abdominal pain, decreased respiratory sounds in both lungs, and bilateral pleural effusion (PE) was found by chest computed tomography (CT) without hepatosplenomegaly. EF is a rare disease in clinical practice. As it involves multiple departments, it is often missed or misdiagnosed as sjögren’s syndrome, rheumatoid arthritis, scleroderma or systemic sclerosis, etc. PE could cause coughing, shortness of breath, difficulty breathing and even respiratory failure. At present, there are few reports about EF combined with PE, which seriously affects the prognosis and quality of life of patients^2^. Therefore, early detection and timely and effective treatment of PE are of great significance for the adjustment of diagnosis and treatment of EF and the prognosis of patients. The purpose of this study was to evaluate the clinical characteristics and related risk factors of EF-PE, so as to guide clinical treatment and improve the prognosis of patients with EF.

## Patients and methods

### Study patients

**Figure 1:**
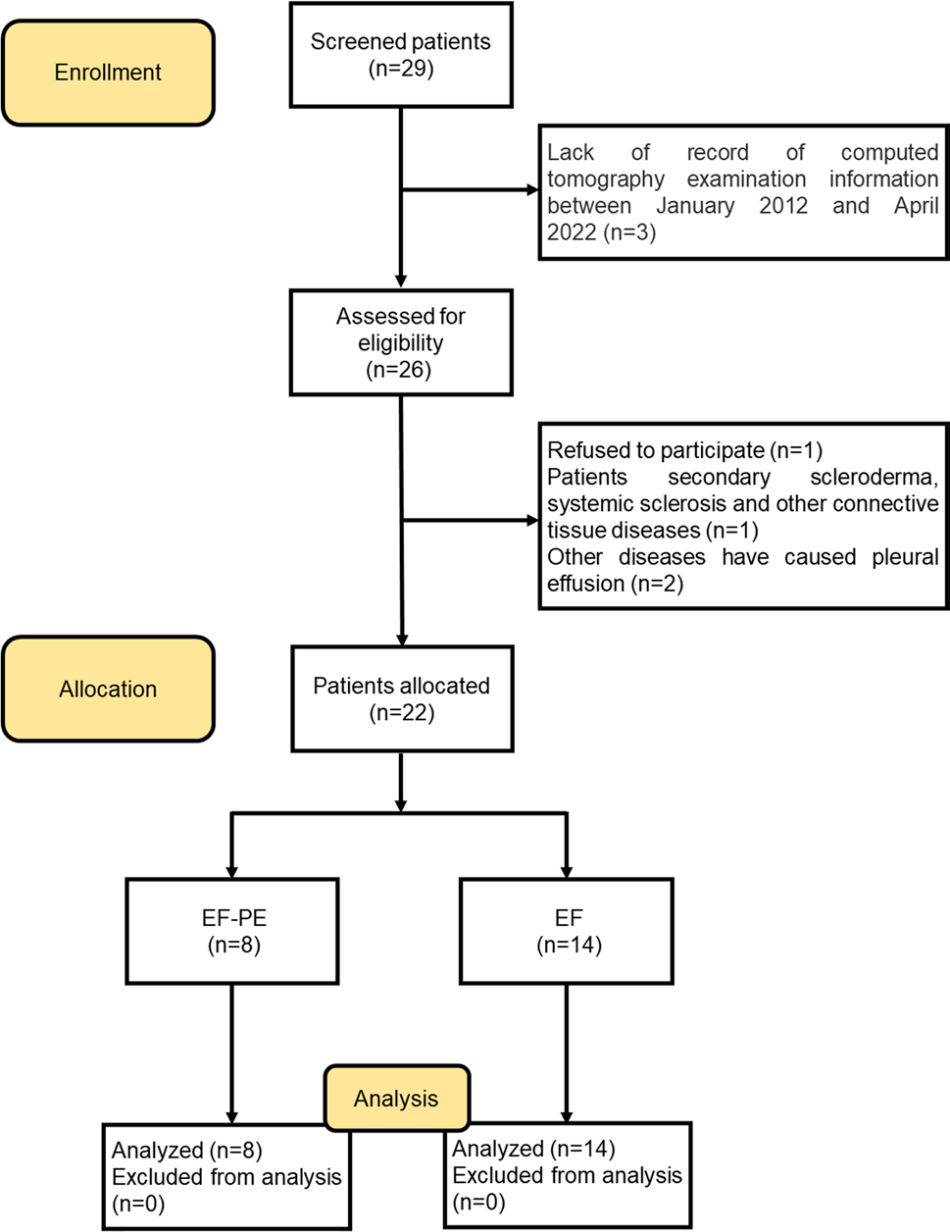
Flow diagram of the study.

This study was approved by the Ethics Review Committee of the Second Xiangya Hospital of Central South University (Ethical Code: LYF2022085), all patients signed an informed consent form and all experiments were performed in accordance with the Declaration of Helsinki. We included 22 patients with EF who were hospitalized in the department of Dermatology, Rheumatology and Respiratory Medicine at the Second Xiangya Hospital of Central South University (Hunan, China) between January 2012 and April 2022. The following inclusion criteria were used for patients with EF: (a) EF was diagnosed in accordance with the diagnostic criteria proposed by Pinal-Fernandez et al. in 2014 and Jinnin et al. in 2018^2,7^; (b) patient data were complete. PE was confirmed by chest CT and ultrasound-guided thoracentesis^8^. And the following patients were excluded: (a) Patients secondary to scleroderma, vascular syndrome, systemic sclerosis and other connective tissue diseases; (b) Diseases with other causes have been confirmed to lead to pleural effusion, such as potential malignancies, congestive heart failure, cirrhosis, tuberculosis and pleurisy, etc.; (c) Chest CT examination was not performed. In this study, patients with EF without PE were used as controls. Patients with PE related symptoms, such as exertive dyspnea or evidence of pleural effusion on chest CT, were excluded. A detailed description of the flow diagram for recruiting voluntary patients can be found in Fig. 1.

### Data collection

After the collection of written informed consent, the demographic characteristics (age, gender, height, weight, smoking history, disease duration), clinical manifestations, concomitant diseases, laboratory data, Biopsy data, chest CT data, and (magnetic resonance imaging, MRI) data of affected sites of all enrolled patients with EF were documented.

### Statistical analysis

SPSS 26.0 software (IBM Corp.) was used to perform all statistical analyses. Continuous variables were described as the mean ± standard deviation (M ± SD) or median (interquartile range), and categorical variables were expressed as the number (percentage). The comparisons of continuous variables were conducted using Student’s *t* test or the Mann–Whitney U test. Categorical variables, including the proportions, were compared using the chi-squared test or Fisher’s exact test. Risk factors were identified by multivariate logistic regression analysis. A* P* value < 0.05 indicated a statistically significant difference.

### Ethical approval

This study was approved by the Ethics Review Committee of the Second Xiangya Hospital of Central South University (Ethical Code: LYF2022085), all patients signed an informed consent form and all experiments were performed in accordance with the Declaration of Helsinki.

## Results

### Demographic characteristics of the two groups

Table 1 shows the demographic characteristics of the 22 patients, including 8 patients with EF-PE (36.36%) and 14 patients with EF (63.63%). Most of the patients with EF-PE were male, with 5 (62.50%).

**Table 1:** Demographic characteristics of the two groups.

Items	EF-PE (n = 8)	EF (n = 14)	*P* value
Age (y), M ± SD	57.75 ± 10.52	47.14 ± 8.58	0.018
Sex M/F, n/n (%/%)	5/3 (62.50%)/(37.50%)	10/4 (71.40%)/(28.60%)	0.667
Disease duration (y)	2.20 ± 1.18	1.23 ± 0.84	0.038
Smoking history, n (%)	7 (87.50%)	10 (71.40%)	0.370
Years of smoking (y)	8.75 ± 5.62	10.57 ± 9.12	0.616
History of overwork	7 (87.50%)	13 (92.90%)	0.679
BMI (kg/m^2^)	21.41 ± 3.07	23.23 ± 3.42	0.228

Comparisons were determined by Student’s *t* test or the Mann–Whitney U test, chi-square test or Fisher’s exact test. *P* < 0.05 was considered statistically significant.

*M* male, *F* female, *BMI* body mass index, *M* ± *SD* mean ± standard deviation.

The age and course of disease in EF-PE group were significantly higher than that in EF group (*P* < 0.05; Table 1). There were no significant differences in gender, smoking history, smoking duration, history of overwork and body mass index (BMI) between 2 groups (*P* > 0.05). Detailed information on patient characteristics is shown in Table 1.

### Clinical manifestations of the two groups

Table 2 shows the clinical manifestations of the two groups. The incidence of fever, weight loss, cough and shortness of breath in EF-PE group was significantly higher than that in EF group (*P* < 0.05; Table 2). There was no significant difference in the incidence of other clinical symptoms between the two groups (*P* > 0.05).

**Table 2:** Clinical manifestations of the two groups.

Items	EF-PE (n = 8)	EF (n = 14)	*P* value
n (%)			
Fever	6 (75.00%)	4 (28.60%)	0.033
Calcareous skin	3 (37.50%)	6 (42.90%)	0.805
Skin erythema	5 (62.50%)	4 (28.60%)	0.119
Skin pigmentation	3 (37.50%)	9 (64.30%)	0.223
Orange peel	4 (50.00%)	9 (64.30%)	0.513
Facial skin sclerosis	3 (37.50%)	6 (42.90%)	0.805
Swelling of the skin	7 (87.50%)	11 (78.60%)	0.593
Skin sclerosis	8 (100.00%)	14 (100.00%)	0.001
Thicken Skin	4 (50.00%)	7 (50.00%)	1.000
Weight loss	6 (75.00%)	4 (28.60%)	0.033
Arthralgia	8 (100.00%)	12 (85.70%)	0.166
Limited joint motion	7 (87.50%)	7 (50.00%)	0.065
Tendon contracture	1 (12.50%)	2 (14.30%)	0.906
Myodynia	4 (50.00%)	7 (50.00%)	1.000
Swelling and sclerosis of limbs	8 (100.00%)	12 (85.70%)	0.166
The skin lesions involved			
Both upper limbs involved	0 (0)	1 (7.10%)	0.335
Both lower limbs involved	2 (25.00%)	2 (14.30%)	0.537
Trunk suffers	3 (37.50%)	5 (35.70%)	0.933
Affected limbs	6 (75.00%)	9 (64.30%)	0.600
Trunk and both upper limbs involved	0 (0)	1 (7.10%)	0.335
Trunk and both lower limbs involved	1 (12.50%)	1 (7.10%)	0.679
Trunk and limbs involved	2 (25.00%)	2 (14.30%)	0.537
Pruritus	4 (50.00%)	3 (21.40%)	0.170
Cough	8 (100.00%)	5 (35.70%)	0.001
Shortness of breath	7 (87.50%)	4 (28.60%)	0.005
Edema	6 (75.00%)	9 (64.30%)	0.600

Comparisons were determined by chi-square test or Fisher’s exact test. *P* < 0.05 was considered statistically significant.

### Laboratory indicators of the two groups

**Figure 2:**
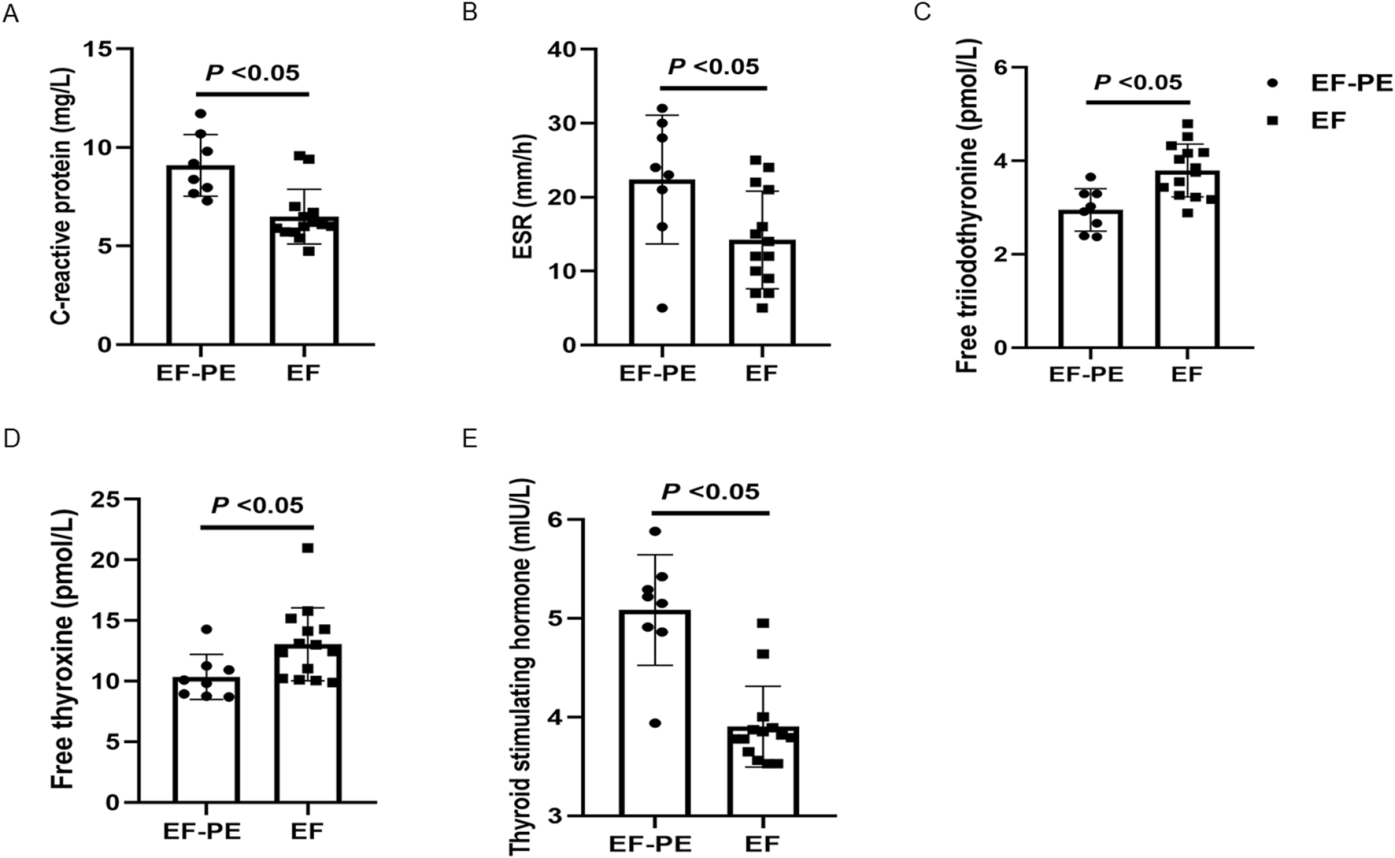
The levels of C-reactive protein, ESR, free triiodothyronine, free thyroxine, and thyroid stimulating hormone in EF-PE and EF groups. The levels of C-reactive protein (**A**), ESR (**B**), free triiodothyronine (**C**), free thyroxine (**D**), and thyroid stimulating hormone (**E**) were compared between EF-PE and EF groups. Comparisons between the two groups were performed by Student’s *t* test or the Mann–Whitney U test. *P* < 0.05 was considered statistically significant.

Table 3 shows the laboratory indicators of the two groups. The levels of C-reactive protein, ESR and thyroid stimulating hormone in EF-PE group were higher than those in EF group, while the levels of free triiodothyronine and free thyroxine were lower than those in EF group, the differences were statistically significant (*P* < 0.05; Table 3 and Fig. 2). There was no significant difference in other laboratory indexes between the two groups (*P* > 0.05). The pleural effusion was aspirated and laboratory tests were performed. The pleural effusion in the eight patients with EF-PE was found to be positive for Rivalta test, accompanied by elevated levels of total protein and adenosine deaminase (normal reference range: 0–24 μ/L). These results indicated that PE associated with EF is exudate. The pleural effusion showed lymphocyte, histocyte and inflammatory cells, mainly eosinophils, but no malignant tumor cells, and the acid-fast stain, Gram stain and bacterial culture were negative. The level of carcinoembryonic antigen was in the normal range (normal reference range: 0–6.5 ng/mL). These results indicate that PE associated with EF is not caused by malignant tumor cells, bacterial infection or mycobacterium tuberculosis.

**Table 3:** Laboratory indicators of the two groups.

Items	EF-PE (n = 8)	EF (n = 14)	*P* value
M ± SD			
White blood cell (× 10^9^/L)	10.74 ± 6.72	8.53 ± 3.80	0.331
Blood platelet (× 10^9^/L)	286.50 ± 90.79	235.07 ± 80.66	0.184
Blood neutrophils (× 10^9^/L)	7.57 ± 6.73	5.56 ± 3.46	0.364
Blood lymphocytes (× 10^9^/L)	2.29 ± 1.44	1.58 ± 0.63	0.125
Blood eosinophils (× 10^9^/L)	0.70 ± 0.38	0.62 ± 0.31	0.571
ALT (IU/L)	15.96 ± 11.66	23.87 ± 24.05	0.396
AST (IU/L)	14.78 ± 2.23	26.70 ± 21.78	0.142
Blood urea nitrogen (mmol/L)	8.83 ± 9.34	4.76 ± 1.56	0.121
Creatinine (μmol/L)	115.91 ± 179.81	74.32 ± 54.89	0.425
Trioxypurine (μmol/L)	307.15 ± 85.92	331.10 ± 75.02	0.502
C-reactive protein (mg/L)	9.09 ± 1.56	6.49 ± 1.38	0.002
ESR (mm/h)	22.37 ± 8.70	14.21 ± 6.60	0.022
C3 (g/L)	1.95 ± 1.19	1.78 ± 0.88	0.710
C4 (g/L)	0.27 ± 0.03	0.26 ± 0.08	0.818
IgA (g/L)	3.94 ± 1.55	4.40 ± 1.68	0.540
IgG (g/L)	19.11 ± 7.71	23.25 ± 6.85	0.207
IgM (g/L)	1.28 ± 0.80	1.77 ± 0.66	0.140
Kalium (mmol/L)	4.05 ± 1.54	3.88 ± 0.35	0.699
Natrium (mmol/L)	140.16 ± 3.39	139.71 ± 2.60	0.732
Calcium (mmol/L)	2.13 ± 0.13	2.11 ± 0.12	0.652
Phosphorus (mmol/L)	1.32 ± 0.22	1.26 ± 0.21	0.514
Total cholesterol (mmol/L)	4.30 ± 0.73	4.10 ± 0.76	0.549
Total cholesterol (mmol/L)	2.17 ± 1.58	2.18 ± 1.29	0.991
D-dimer (μg/mL)	1.72 ± 1.11	2.41 ± 0.97	0.147
Glucose (mmol/L)	4.92 ± 1.05	5.22 ± 0.69	0.433
Free triiodothyronine (pmol/L)	2.94 ± 0.45	3.79 ± 0.56	0.002
Free thyroxine (pmol/L)	10.34 ± 1.86	13.04 ± 3.02	0.034
Thyroid stimulating hormone (mIU/L)	5.08 ± 0.56	3.90 ± 0.41	0.000
Lactic dehydrogenase (μ/L)	203.65 ± 39.29	208.18 ± 27.41	0.753
Creatine kinase (μ/L)	39.93 ± 12.26	43.56 ± 6.11	0.362
NT-proBNP (pg/mL)	42.63 ± 5.34	39.61 ± 5.52	0.411
Pleural effusion			
Colour, n (%)	8		
Red	3 (37.50%)		
Yellow	5 (62.50%)		
Transparence, n (%)	8		
Clearness	2 (25.00%)		
Turbidity	6 (75.00%)		
Rivalta test positive, n (%)	8 (100%)		
Total cell count (× 10^6^/L)	2087.37 ± 168.76		
White cell count (× 10^6^/L)	351.50 ± 65.94		
Eosinophils positive, n (%)	8 (100%)		
Histocyte positive, n (%)	8 (100%)		
Lymphocyte positive, n (%)	8 (100%)		
Malignant cell positive	0		
Acid-fast stain positive	0		
Bacterial culture positive	0		
Gram stain positive	0		
Adenosine deaminase (μ/L)	32.81 ± 4.41		
Total protein (g/L)	42.58 ± 5.69		
Lactic dehydrogenase (μ/L)	272.91 ± 33.84		
Carcino-embryonic antigen (ng/mL)	1.10 ± 0.63		

Comparisons were determined by Student’s *t* test or the Mann–Whitney U test. *P* < 0.05 was considered statistically significant.

*ESR* erythrocyte sedimentation rate, *M* ± *SD* mean ± standard deviation, *NT-proBNP* N-terminal probrain natriuretic peptide.

### Concomitant diseases of the two groups

Table 4 shows the concomitant diseases of the two groups. The incidence of pulmonary infection, hypothyroidism, hydronephrosis and kidney stones in EF-PE group was higher than that in EF group (*P* < 0.05; Table 4). There was no significant difference in the incidence of other complications between the two groups (*P* > 0.05).

**Table 4:** Concomitant diseases of the two groups.

Items	EF-PE (n = 8)	EF (n = 14)	*P* value
n (%)			
Hypertension	3 (37.50%)	5 (35.70%)	0.933
Pulmonary infection	5 (62.50%)	2 (14.30%)	0.020
Herniation of cervical disc	0 (0)	3 (21.40%)	0.084
Protrusion of lumbar intervertebral disc	3 (37.50%)	1 (7.10%)	0.080
Hypothyroidism	5 (62.50%)	1 (7.10%)	0.005
Fatty liver	3 (37.50%)	3 (21.40%)	0.421
Chronic Gastritis	1 (12.50%)	1 (7.10%)	0.679
Hydronephrosis	6 (75.00%)	3 (21.40%)	0.013
Kidney stone	5 (62.50%)	2 (14.30%)	0.020

Comparisons were determined by chi-square test or Fisher’s exact test. *P* < 0.05 was considered statistically significant.

### Histopathological examination results of the two groups

Table 5 shows the histopathological examination results of the two groups. The swelling rate of small vascular endothelial cells in EF-PE group was higher than that in EF group (*P* < 0.05; Table 5). There was no significant difference in the incidence of other histopathological examination results between the two groups (*P* > 0.05).

**Table 5:** Histopathology of the two groups.

Items	EF-PE (n = 8)	EF (n = 14)	*P* value
n (%)			
Lymphocytes infiltration	7 (87.50%)	14 (100.00%)	0.147
Plasmocytes infiltration	6 (75.00%)	11 (78.60%)	0.848
Eosinophils infiltration	6 (75.00%)	8 (57.10%)	0.395
Hard patchy plaques	4 (50.00%)	4 (28.60%)	0.317
Fascial thickening	6 (75.00%)	10 (71.40%)	0.856
Hyperplasia of fascia	6 (75.00%)	7 (50.00%)	0.243
Swollen small vascular endothelial cells	4 (50.00%)	1 (7.10%)	0.021
Intima thickening	5 (62.50%)	9 (64.30%)	0.933
Collagen fiber bundles increase	1 (12.5%)	0 (0)	0.147
Fibrotic superficial dermis and epidermis	4 (50.00%)	4 (28.60%)	0.317

Comparisons were determined by chi-square test or Fisher’s exact test. *P* < 0.05 was considered statistically significant.

### MRI of the affected sites and chest CT of the two groups

Table 6 shows the MRI of the affected sites and chest CT of the two groups. The positive rate of chest CT consolidation shadows in EF-PE group was higher than that in EF group (*P* < 0.05; Table 6). There was no significant difference in the positive rate of other imaging examinations between the two groups (*P* > 0.05).

**Table 6:** MRI and chest CT examination results of the affected sites in both groups.

Items	EF-PE (n = 8)	EF (n = 14)	*P* value
n (%)			
MRI results of affected sites			
T1-weighted images	6 (75.00%)	10 (71.40%)	0.856
T2-weighted images	6 (75.00%)	10 (71.40%)	0.856
Strengthening	6 (75.00%)	10 (71.40%)	0.856
Mild strengthening	2 (25.00%)	5 (35.70%)	0.794
Moderate strengthening	4 (50.00%)	5 (35.70%)	
Chest CT results			
Ground-glass opacity	5 (62.50%)	3 (21.40%)	0.054
Consolidation shadows	6 (75.00%)	4 (28.60%)	0.033
Interlobular septa thickened	4 (50.00%)	3 (21.40%%)	0.170
Hydropericardium	5 (62.50%)	7 (50.00%)	0.570
Pleural thickening	4 (50.00%)	2 (14.30%)	0.073

Comparisons were determined by chi-square test or Fisher’s exact test. *P* < 0.05 was considered statistically significant.

*CT* computed tomography, *MRI* magnetic resonance imaging.

### Multivariate logistic regression analysis of risk factors for EF happening PE based on clinical parameters and sociodemographic characteristics

Table 7 shows the multivariate logistic regression analysis of risk factors for EF happening PE based on clinical parameters and sociodemographic characteristics of the two groups.

**Table 7:** Multivariate logistic regression analysis of risk factors for EF happening PE based on clinical parameters and sociodemographic characteristics.

Variable	*P* value	*OR*	95%*CI*
Age	0.042	1.136	1.005–1.284
Fever	0.046	7.500	1.039–54.116
Shortness of breath	0.019	17.500	1.596–191.892
C-reactive protein	0.012	2.885	1.264–6.589
ESR	0.039	1.162	1.007–1.339
Free triiodothyronine	0.026	0.029	0.001–0.651
Free thyroxine	0.049	0.553	0.306–0.999
Thyroid stimulating hormone	0.006	33.921	1.747–418.838
Pulmonary infection	0.029	10.000	1.260–79.339
Hypothyroidism	0.015	21.667	1.802–260.574
Hydronephrosis	0.022	11.000	1.420–85.201
Kidney stone	0.029	10.000	1.260–79.339
Swollen small vascular endothelial cells	0.041	13.000	1.109–152.351
Consolidation shadows	0.046	7.500	1.039–54.116

Values were expressed as odds ratio (OR) and 95% confidence interval (CI). Risk factors associated with EF-PE were determined by multivariate logistic regression analysis. *P* < 0.05 was considered statistically significant.

*ESR* erythrocyte sedimentation rate, *95% CI* 95% confidence interval, *OR* odds ratio.

According to the sociodemographic characteristics, patients who were older were more likely to occur PE, with an *OR* of 1.136 (95%*CI* = 1.005–1.284, *P* = 0.042). According to the clinical parameters, fever (*OR* = 7.500, 95%*CI* = 1.039–54.116, *P* = 0.046), shortness of breath (*OR* = 17.500, 95%*CI* = 1.596–191.892, *P* = 0.019), C-reactive protein (*OR* = 2.885, 95%*CI* = 1.264–6.589, *P* = 0.012), ESR (*OR* = 1.162, 95%*CI* = 1.007–1.339, *P* = 0.039), thyroid stimulating hormone (*OR* = 33.921, 95%*CI* = 1.747–418.838, *P* = 0.006), pulmonary infection (*OR* = 10.000, 95%*CI* = 1.260–79.339, *P* = 0.029), hypothyroidism (*OR* = 21.667, 95% *CI* = 1.802–260.574, *P* = 0.015), hydronephrosis (*OR* = 11.000, 95%*CI* = 1.420–85.201, *P* = 0.022), kidney stone (*OR* = 10.000, 95%*CI* = 1.260–79.339, *P* = 0.029), swollen small vascular endothelial cells (*OR* = 13.000, 95%*CI* = 1.109–152.351, *P* = 0.041), and chest CT consolidation shadows (*OR* = 7.500, 95%*CI* = 1.039–54.116, *P* = 0.046) were identified as risk factors for happening PE in patients with EF, while free triiodothyronine (*OR* = 0.029, 95%*CI* = 0.001–0.651, *P* = 0.026) and free thyroxine (*OR* = 0.553, 95%*CI* = 0.306–0.999, *P* = 0.049) were identified as protective factors against PE in patients with EF, with low levels of free triiodothyine and free thyroxine more likely to develop PE (Table 7).

## Discussion

EF, first reported in 1974, is a rare clinical disease characterized by diffuse inflammation of the fascia and swelling and hardening of the skin. EF can cause diseases of the blood system, autoimmune diseases, lung and pleural diseases. It has been reported that patients with EF may develop bilateral pleural effusion, causing cough and dyspnea, which seriously affects the prognosis and quality of life of the patients^6^. However, the early prevalence of EF-PE may be underestimated due to the lack of obvious clinical symptoms in the early stage. Therefore, it is of great clinical significance to investigate the incidence of EF-PE and discuss the risk factors for its occurrence. In this study, 22 patients with EF by chest CT examination found that there are 8 cases (36.36%) complicated with PE, this is the first report of 8 patients with EF-PE, compared with the case before the medical record report, this may be related to sample source, sample size, the extensive examination of chest CT in EF and clinicians in improving the sensitivity of the PE.

At present, studies have found that the onset age of EF is mainly 40–50 years old, and there are also reports of young and old people^9^. In this study, the age of patients with EF-PE was higher than that of patients with EF, which was consistent with the results of previous case reports^6,10^. In addition, in this study, the disease course of patients with EF-PE is higher than that of patients with EF, and it has been reported that 10%-20% of patients will automatically resolve after 2–5 years of disease, and if complicated with PE, the disease course may be prolonged^11^. Multivariate logistic regression analysis showed that advanced age was a risk factor for PE in patients with EF. The possible reason was that elderly patients were vulnerable to infiltration of inflammatory cells due to weakened immune system defense, and inflammatory cells and inflammatory mediators would cause vasoconstriction and increased resistance, leading to the occurrence of PE. It is also possible that patients with EF are not detected early enough, resulting in an older age at diagnosis of EF-PE.

EF-PE is a rare disease that often involves multiple clinical departments. It has a variety of clinical symptoms and is difficult to detect in time. In this study, the incidence of fever, weight loss, cough and shortness of breath was higher in patients with EF-PE than that in patients with EF, which was consistent with the dyspnea reported by Killen et al. in patients with EF complicated with PE^10^, but fever was not reported in this case. patients with EF complicated with PE often have poor appetite, resulting in weight loss, cough, wheezing, shortness of breath and other symptoms, and even dyspnea. Multivariate logistic regression analysis showed that fever and shortness of breath were risk factors for PE in patients with EF. When patients with EF showed symptoms of fever, wheezing and shortness of breath, they should be aware of the possibility of PE.

Peripheral blood eosinophils, C-reactive protein and ESR are elevated in most patients with EF^12,13^. In this study, the pleural effusion in the patients with EF-PE was found to be positive for Rivalta test, and was associated with elevated levels of total protein and adenosine deaminase, indicating that the pleural effusion was an exudate in the patients with EP-PE. In addition, inflammatory cells were found in pleural effusion, mainly eosinophils, but no pathogenic bacteria, mycobacterium tuberculosis or malignant tumor cells. This is consistent with Killen et al. ‘s discovery of eosinophilic inflammatory cells in pleural effusion in patients with EF^10^. In this study, eosinophils in peripheral blood of patients with EF increased, but there was no statistically significant difference between EF-PE and patients with EF. Lebeaux et al. reported that not all patients would have increased eosinophils, 69% of patients in acute stage will be the increase in the number of eosinophils and as the disease development, under the condition of without intervention, eosinophils could descend to reference range, eosinophil levels and disease activity and severity is not parallel^9^. In this study, the C-reactive protein and ESR of patients with EF-PE were higher than those of patients with EF, and multivariate logistic regression analysis showed that C-reactive protein and ESR were risk factors for happening EF-PE. Interestingly, this study found for the first time that the level of thyroid stimulating hormone in patients with EF-PE was higher than that in patients with EF, while the level of free triiodothyronine and free thyroxine was lower than that in patients with EF. Hemorrhagic pleural effusion and hemorrhagic pericardial effusion have been reported in untreated hypothyroidism caused by pneumonia^14^. No other conditions causing hypothyroidism, such as severe infection, malignancy, or trauma, were found in the patients with EF and EF-PE in this study. Unfortunately, patients are not willing to undergo thyroid histopathological tests. Meanwhile, multivariate logistic regression analysis found that thyroid stimulating hormone was a risk factor for occurring EF-PE. Free triiodothyronine and free thyroxine were protective factors against EF-PE. When the above laboratory indicators are abnormal, the possible complications of EF should be considered in time, so as to achieve early diagnosis and treatment.

The main clinical features of EF are symmetrical, progressive sclerosclerosis of the limbs, joint pain, limited movement, and orange skin sign. Histopathologic examination of the affected sites may show infiltration of lymphocytes or plasma cells, accompanied by increased eosinophils and fascia hyperplasia and thickening^13^. In this study, all the 22 patients were examined by skin or muscle biopsy and found infiltration of lymphocytes and plasma cells, with or without eosinophil infiltration, fascia hyperplasia and intima thickening, which were consistent with previous reports. In addition, our study found that the incidence of small vascular endothelial cell swelling in patients with EF-PE was higher than that in patients with EF, and multivariate logistic regression analysis showed that small vascular endothelial cell swelling was a risk factor for happening EF-PE. It may be because inflammatory cells and mediators invade small vascular endothelial cells, resulting in endothelial cell injury, smooth muscle cell proliferation, increased vascular pressure, and promote the occurrence of PE. In this study, the MRI T1 and T2-weighted images of patients with EF at the affected site showed high signals, indicating the thickening of the affected fascia accompanied by mild to moderate strengthening, which was consistent with literature reports^15^. MRI has been recommended to track the course of disease in treated patients. However, in the absence of prospective studies, there are no rigorous criteria to assess the response to MRI therapy, nor has the optimal follow-up time for MRI therapy in EF been determined^16^.

EF is associated with systemic diseases such as hematological disease, neoplastic disease, lung and pleural disease^4,17^. In this study, the incidence of pulmonary infection, hypothyroidism, hydronephrosis, and kidney stones was higher in patients with EF-PE than that in patients with EF, while the incidence of other complications showed no statistical difference between the two groups. In the chest CT results of this study, the positive rate of consolidation shadows was higher in patients with EF-PE than that in patients with EF. Multivariate logistic regression analysis showed that pulmonary infection, hypothyroidism, hydronephrosis, kidney stones and chest CT consolidation shadows were risk factors for PE in patients with EF. Hypothyroidism, kidney seeper, and kidney stones can weaken the immune system’s defenses of patients, chest CT consolidation shadows often hint associated with pulmonary infection, accompanied by infiltration of inflammatory cells and mediators, and at the same time cause the release of a variety of proinflammatory factor, which can cause vasculitis causing vascular endothelial cell damage, make higher blood pressure and permeability increases, eventually occurring PE^18,19^.

There were some limitations of this study: (a) In the baseline data of this study, the average age of patients with EF-PE was 10 years older and the average course of disease was 1 year longer. Since EF is a rare disease with a small sample size, it may be a confounding factor for the baseline risk of PE. Future studies will strive to include more cases and try to control confounding factors. (b) This is a cross-sectional descriptive study; therefore, we cannot draw conclusions about the direction of causation. (c) As eosinophilic fasciitis with pleural effusion is a rare disease, therefore, additional cases and follow-up of patients need to be collected for further validation. (d) The mechanism of eosinophilic fasciitis associated with pleural effusion remains unexplained and needs further investigation.

## Conclusion

This is the first study to report the incidence of EF-PE and to investigate the risk factors associated with it. In this study, the incidence of EF-PE was relatively high (36.36%), and advanced age, high C-reactive protein, high ESR, high thyroid stimulating hormone, low free triiodothyronine, low free thyroxine, the high incidence of fever, shortness of breath, pulmonary infection, hydronephrosis, kidney stones, swollen small vascular endothelial cells and chest CT consolidation shadows suggested that the risk of happening PE in patients with EF was significantly increased. More attention should be paid to patients with EF with these risk factors in clinical diagnosis and treatment.

## Data Availability

The data used and analyzed in this study are available from the corresponding author on reasonable request; E-mail: ouyangruoyun@csu.edu.cn.
